# Case Report: High altitude, young lives: unmasking pulmonary hypertension in two unique cases

**DOI:** 10.3389/fmed.2025.1719635

**Published:** 2025-12-01

**Authors:** Fuguo Gao, Rui Liu, Yi Sun, He Huang, Siyang Zuo, Chengxing Yang, Po Ma, Bin Li, Yan Hou, Qingliang Xue

**Affiliations:** 1 Department of Pulmonary and Critical Care Medicine, The 940th Hospital of Joint Logistics Support Force of Chinese People’s Liberation Army, Lanzhou, China; 2 Department of Plateau Medicine, The 940th Hospital of Joint Logistics Support Force of Chinese People’s Liberation Army, Lanzhou, China; 3 Department of Cardio-renal Medicine, The 944th Hospital of Joint Logistics Support Force of Chinese People’s Liberation Army, Jiuquan, China; 4 Department of Ophthalmology, The 940th Hospital of Joint Logistics Support Force of Chinese People’s Liberation Army, Lanzhou, China; 5 Department of Neurology, The 940th Hospital of Joint Logistics Support Force of Chinese People’s Liberation Army, Lanzhou, China; 6 Department of Ultrasound Diagnostics, The 940th Hospital of Joint Logistics Support Force of Chinese People’s Liberation Army, Lanzhou, China; 7 Department of Interventional Therapy, The 940th Hospital of Joint Logistics Support Force of Chinese People’s Liberation Army, Lanzhou, China; 8 Department of Radiology, The 940th Hospital of Joint Logistics Support Force of Chinese PLA, Lanzhou, Gansu, China

**Keywords:** high altitude pulmonary hypertension, high altitude heart disease, hypoxia, right heart dysfunction, pulmonary edema

## Abstract

**Background:**

The high-altitude environment characterized by hypobaric hypoxia can cause significant damage to the cardiovascular system, particularly vascular endothelial function, and is a significant trigger for acute and chronic mountain sickness. High-altitude pulmonary hypertension (HAPH) is a serious complication induced by prolonged exposure to high altitude, characterized by abnormally elevated pulmonary artery pressure and increased right heart load, which can progress to right heart failure and be life-threatening. This article aims to enhance clinicians’ understanding of this disease through two cases of HAPH in young males.

**Case description:**

Case 1 was a 21-year-old male who developed progressively worsening chest pain, shortness of breath, and amaurosis fugax after living at an altitude of 4800 meters for 4 months. Case 2 was a 20-year-old male who experienced exertional dyspnea accompanied by amaurosis fugax and a brief loss of consciousness after 18 months of residence at 4,300 meters. Both patients were previously healthy with normal cardiopulmonary function before moving to high altitude. Physical examination upon presentation revealed significant hypoxemia (oxygen saturation 80 and 82% on room air, respectively). Echocardiography in both cases clearly demonstrated right atrial and right ventricular enlargement, moderate tricuspid regurgitation, with estimated systolic pulmonary artery pressures (sPAP) as high as 55 mmHg and 56 mmHg, respectively. Chest CT confirmed right heart enlargement and main pulmonary artery dilation. After systematic evaluation excluded other etiologies, HAPH was diagnosed.

**Discussion:**

The core pathophysiology of HAPH is hypoxia-induced pulmonary vasoconstriction and remodeling. These cases demonstrate that even for young, healthy individuals, prolonged exposure to extremely high altitudes can lead to severe pulmonary hypertension and right ventricular dysfunction. Diagnosis requires a combination of high-altitude exposure history, clinical symptoms, evidence of hypoxemia, and echocardiographic findings. The most fundamental treatment for diagnosed HAPH patients is removal from the high-altitude hypoxic environment, making early recognition and intervention crucial.

**Conclusion:**

The high-altitude environment poses a serious threat to the cardiovascular systems of susceptible individuals. For patients with a history of high-altitude residence who present with relevant symptoms, clinicians should maintain a high index of suspicion for HAPH and conduct timely screening and diagnosis to prevent adverse outcomes.

## Introduction

The high-altitude environment (typically defined as > 2,500 m above sea level) attracts a large number of travelers, migrant workers, and long-term residents annually. It is estimated that over 80 million people permanently reside above 2,500 m worldwide (1). However, the extreme environment of hypobaric hypoxia presents a series of physiological challenges to the unacclimatized body, often inducing various high-altitude illnesses (2). At altitudes exceeding 4,000 m, significant damage can occur to the human cardiovascular system, particularly vascular endothelial function (3), manifesting as attenuated exercise-induced vasodilation and persistently elevated basal sympathetic nerve activity (4).

Acute exposure to high altitude can trigger high-altitude illnesses (HAIs), including acute mountain sickness (AMS), high-altitude cerebral edema (HACE), and high-altitude pulmonary edema (HAPE). For long-term residents, persistent stimulation from chronic hypoxia may lead to the development of chronic mountain sickness (CMS) (1). Hypoxia is a potent pulmonary vasoconstrictor, leading to significant increases in pulmonary artery pressure (Ppa) and pulmonary vascular resistance (PVR) (5). If the pulmonary hypertensive state persists, it causes a continuous increase in right ventricular afterload, potentially progressing to right ventricular hypertrophy, dilation, and even right heart failure, seriously threatening life (6, 7). This condition, induced by prolonged high-altitude exposure and excluding other known causes, is defined as high-altitude pulmonary hypertension (HAPH), an important manifestation of chronic mountain sickness.

This paper reports two cases of severe pulmonary hypertension in young males after residing at altitudes above 4,300 m. Both were previously healthy with no underlying cardiopulmonary diseases. Their disease progression highlights the potential severe cardiovascular impact of the high-altitude environment on susceptible individuals. By analyzing their clinical characteristics and imaging findings, we aim to enhance clinicians’ awareness of HAPH, a relatively rare but critical disease, and emphasize the importance of early recognition and intervention to prevent adverse outcomes due to misdiagnosis or delayed treatment.

## Case description

### Case 1

A 21-year-old male presented with “intermittent chest pain for 4 months, worsening with shortness of breath for 2 weeks.” Four months prior, after ascending to a 4,800-m plateau, the patient experienced precordial needle-like pain with low frequency, for which he did not seek treatment. Symptoms worsened over the past 2 weeks, with chest pain frequency increasing to 3–4 times per week, accompanied by chest tightness and shortness of breath triggered by mild activity. During this period, he experienced two episodes of amaurosis fugax, which resolved spontaneously with rest, without loss of consciousness. He reported a recent weight loss of approximately 2.5 kg. Further history revealed that within the first week at high altitude, he had experienced severe headaches relieved by ibuprofen. He used supplemental oxygen intermittently for 2–3 h daily during his stay. Past medical history was unremarkable, with a history of regular exercise. Pre-altitude ECG and echocardiography were normal. No history of infectious diseases, special medication use, family history of heart disease, smoking, or alcohol consumption. The patient denies any history of sojourning at high altitudes, and both parents are native to lowland regions.

#### Vital signs

Temperature 36.5°C, pulse 62 beats/min, respiratory rate 19 breaths/min, blood pressure 116/69 mmHg, oxygen saturation (SpO_2_) 80% on room air. Body mass index 21.15 kg/m^2^. He was conscious, ambulatory, with cyanotic lips. No precordial bulge or abnormal pulsations were noted. Heart rate 63 beats/min, regular rhythm, strong heart sounds, the second heart sound at the pulmonary valve area (P_2_) was greater than that at the aortic area (A_2_). No pathological murmurs, pericardial friction rubs, or knocking sounds were heard over the valve areas. Clear breath sounds bilaterally, soft abdomen, no edema in the lower extremities.

#### Laboratory tests

Arterial blood gas analysis on room air (temperature 36.8°C): pH 7.422, PaCO_2_ 29.0 mmHg, PaO_2_ 52.9 mmHg, Anion Gap (AG) 15.8 mmol/L. Complete blood count: White blood cell count (WBC) 10.3 × 10^9^/L, Red blood cell count (RBC) 8.21 × 10^12^/L, Hemoglobin (Hb) 215 g/L. Liver function: Alanine aminotransferase (ALT) 61.9 U/L, Aspartate aminotransferase (AST) 42.2 U/L, Creatine kinase (CK) 76.5 U/L, Creatine kinase-MB (CK-MB) 40.6 U/L. Low-density lipoprotein cholesterol (LDL-C) 3.14 mmol/L. Troponin I (cTnI), electrolytes, coagulation profile, D-dimer, renal function, and procalcitonin were within normal limits. The 6-min walk distance was 420 m.

#### Imaging and functional studies

**FIGURE 1:**
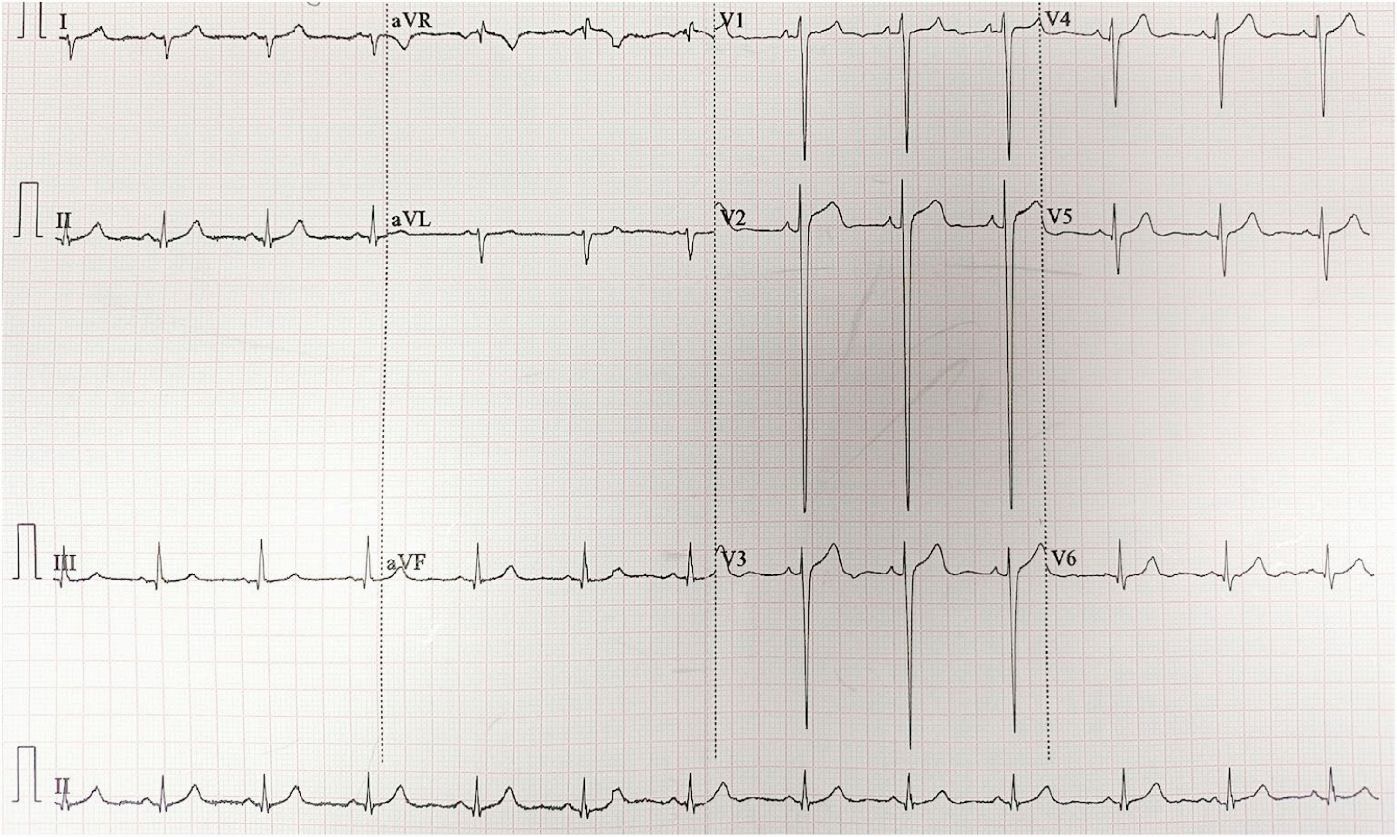
Electrocardiogram recorded at high altitude during the evaluation for HAPH.

**FIGURE 2:**
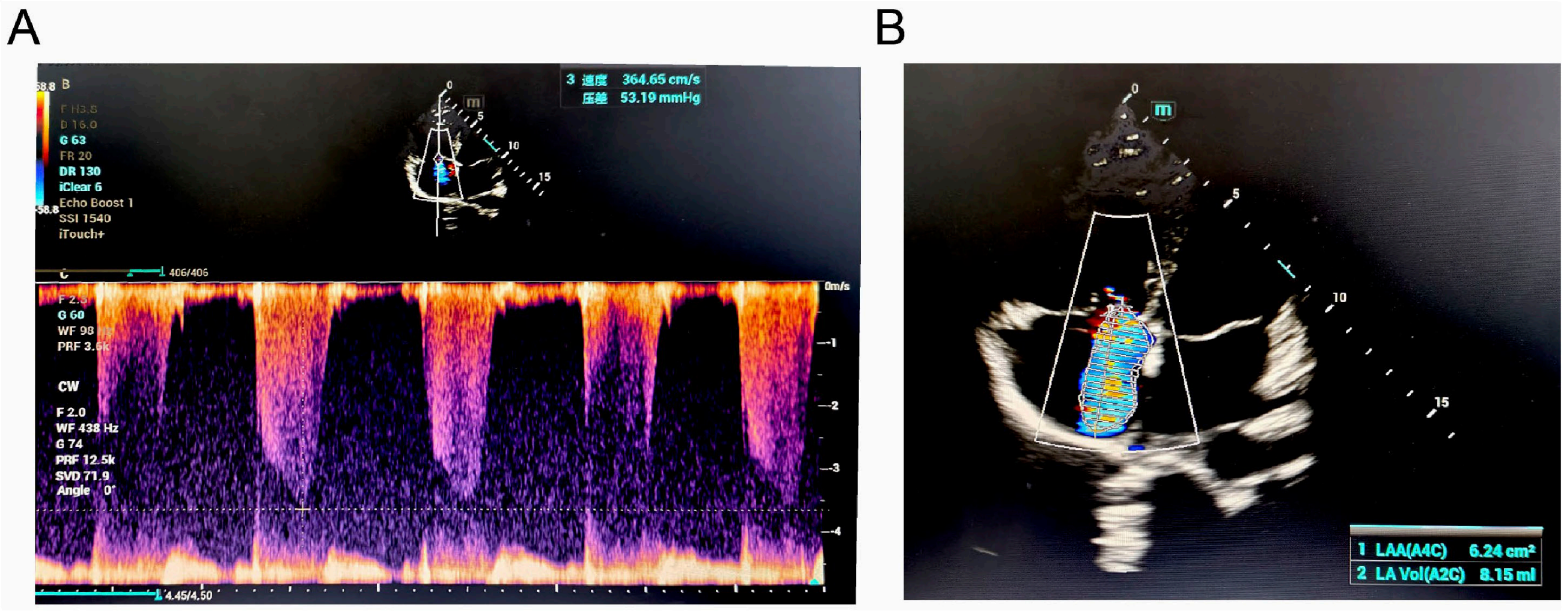
Echocardiographic findings of the patient. **(A)** Continuous-wave Doppler spectrum showing tricuspid regurgitation. **(B)** Apical four-chamber view demonstrating significant tricuspid regurgitation (TR).

**FIGURE 3:**
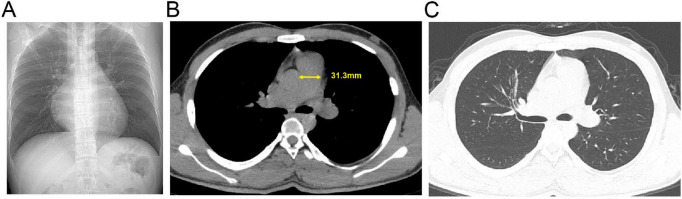
Admission chest computed tomography (CT) of the patient with pulmonary hypertension. **(A)** Axial non-contrast image. **(B)** Mediastinal window view; the yellow solid line indicates the transverse diameter of the main pulmonary artery. **(C)** Lung window view.

ECG showed sinus rhythm, right axis deviation, rS pattern in leads V1-V4 with R/S ratio < 1, and clockwise rotation, suggesting possible right ventricular enlargement (Figure 1). Echocardiography revealed right atrial and right ventricular enlargement (RV anterior-posterior diameter 35 mm; RA left-right diameter 43 mm), moderate tricuspid regurgitation with a maximum regurgitant velocity of 4.6 m/s, estimating a systolic pulmonary artery pressure (sPAP) of approximately 55 mmHg, and the mean pulmonary artery pressure (mPAP) was calculated to be 36 mmHg. The main pulmonary artery was widened to 28 mm. These findings are consistent with high-altitude heart disease (Figure 2). Chest CT indicated cardiomegaly, predominantly affecting the right heart, with the widest point of the main pulmonary artery measuring approximately 3.13 cm (Figure 3).

The patient returned to an area below 1,500 m within 48 h and received no pharmacological or other specific interventions. A follow-up transthoracic echocardiogram performed 6 days later showed an estimated sPAP of approximately 47 mmHg and mPAP of 31 mmHg. One month later, repeat echocardiography demonstrated complete normalization of pulmonary artery pressures.

### Case 2

A 20-year-old male presented with “intermittent exertional dyspnea for 5 months, worsening with amaurosis fugax for 1 week.” The patient had ascended to a 4,300-m plateau 18 months prior. Five months before presentation, he developed intermittent dyspnea triggered by mild physical activity, for which he did not seek treatment. Symptoms persisted. One week prior, after routine training, he experienced an episode of amaurosis fugax accompanied by a brief loss of consciousness (lasting seconds), without convulsions or foaming at the mouth. He regained consciousness spontaneously, feeling chest tightness and shortness of breath, which relieved with low-flow oxygen (2 L/min). No significant weight change was reported. Further history revealed no symptoms of acute mountain sickness upon initial ascent; he did not use supplemental oxygen during his stay. He returned to the plain areas for half a month’s rest and re-ascended to the plateau 10 days before presentation. Past medical history included unspecified “hypoglycemia” and regular exercise. Pre-altitude ECG and echocardiography were normal. No history of infectious diseases, special medication use, family history of heart disease, smoking, or alcohol consumption. The patient denies any history of sojourning at high altitudes, and both parents are native to lowland regions.

#### Vital signs

Temperature 36.5°C, pulse 76 beats/min, respiratory rate 19 breaths/min, blood pressure 123/69 mmHg, SpO_2_ 82% on room air. Body mass index 23.62 kg/m^2^. He was conscious, walked into the hospital, and was cooperative. Lips were not cyanotic. No precordial bulge. Heart rate 76 beats/min, regular rhythm, strong heart sounds, P_2_ > A_2_. No pathological murmurs, pericardial friction rubs, or knocking sounds were heard over the valve areas. Clear breath sounds bilaterally, soft abdomen, no edema in the lower extremities. Neurological examination was normal. The 6-min walk distance was 550 m.

#### Laboratory tests

Arterial blood gas analysis on room air (temperature 36.8°C): pH 7.402, PaCO_2_ 27.7 mmHg, PaO_2_ 56.0 mmHg, AG 17.5 mmol/L. Complete blood count: WBC 5.4 × 10^9^/L, RBC 5.77 × 10^12^/L, Hb 180 g/L. Liver function, uric acid, myocardial enzyme profile, electrolytes, coagulation profile, D-dimer, renal function, and procalcitonin were within normal limits.

#### Imaging and functional studies

**FIGURE 4:**
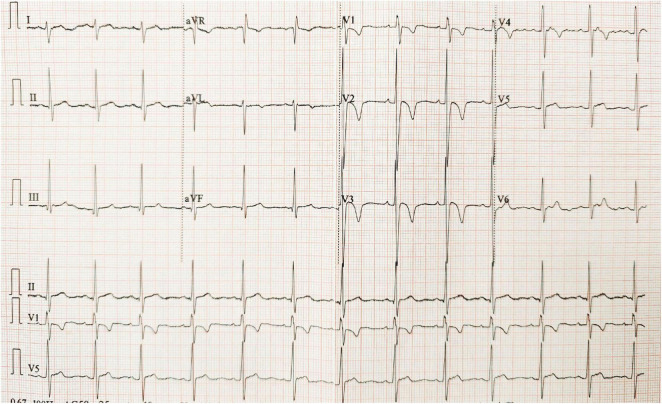
Electrocardiogram recorded at high altitude during the evaluation for HAPH.

**FIGURE 5:**
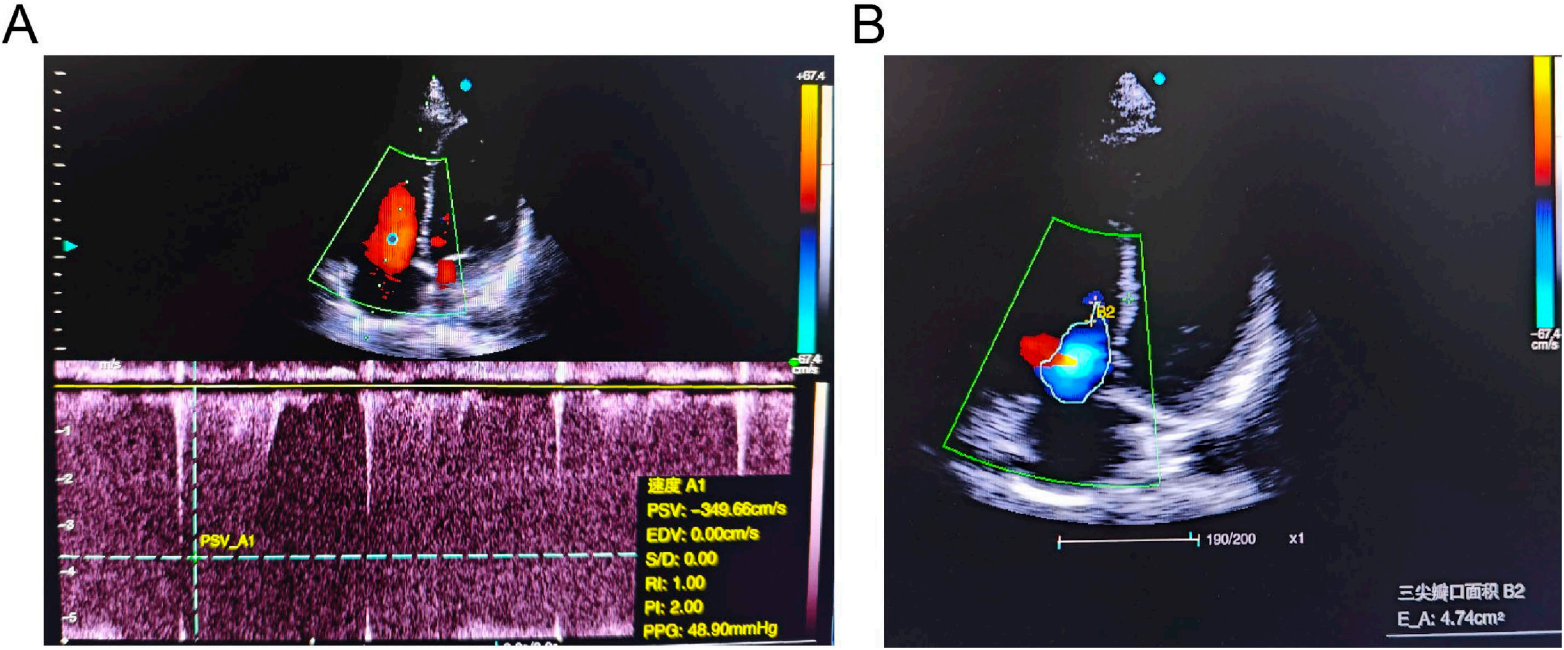
Echocardiographic findings of the patient. **(A)** Continuous-wave Doppler spectrum showing tricuspid regurgitation. **(B)** Apical four-chamber view demonstrating significant tricuspid regurgitation (TR).

**FIGURE 6:**
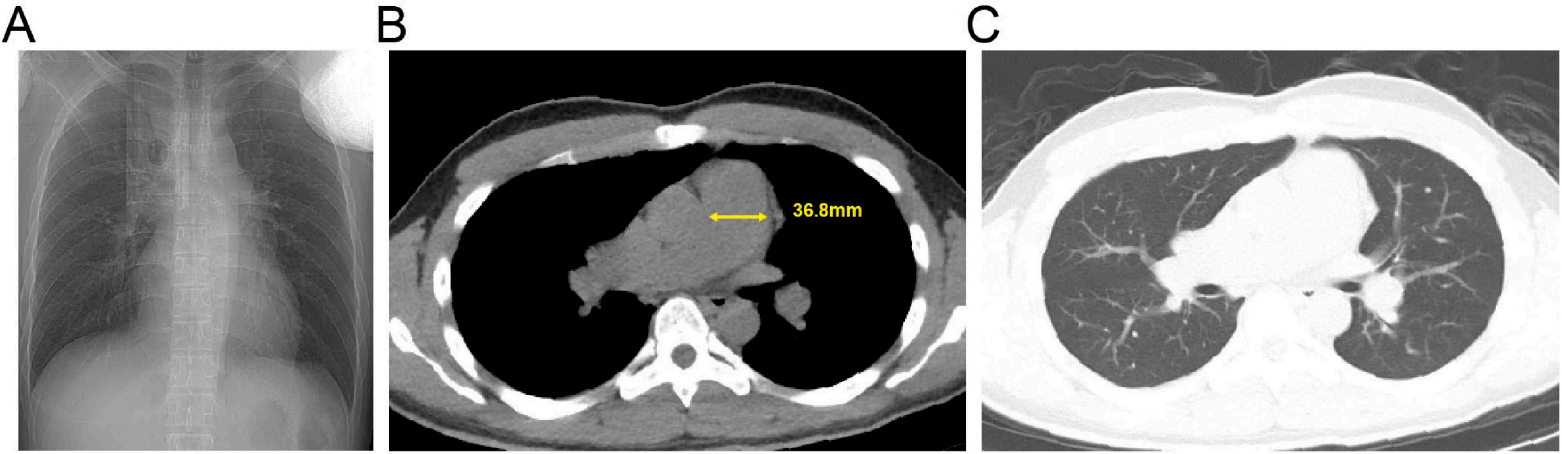
Admission chest computed tomography (CT) of the patient with pulmonary hypertension. **(A)** Axial non-contrast image. **(B)** Mediastinal window view; the yellow solid line indicates the transverse diameter of the main pulmonary artery. **(C)** Lung window view.

ECG showed sinus rhythm, right axis deviation, RS pattern in V1-V2, deep S waves in V4-V6, and asymmetric T-wave inversion in V1-V4, suggesting right ventricular strain (Figure 4). Echocardiography revealed right atrial and right ventricular enlargement (right ventricular transverse diameter 38 mm; right atrial diameter 45 mm), along with moderate tricuspid regurgitation. The peak regurgitant velocity was 4.3 m/s, yielding an estimated sPAP of approximately 56 mmHg and mPAP of about 36 mmHg. Pulmonary artery dilation (28 mm) was also noted. These findings are consistent with high-altitude heart disease (Figure 5). Carotid ultrasound showed no abnormalities. Chest CT indicated cardiomegaly, predominantly affecting the right heart, with the widest point of the pulmonary artery measuring approximately 3.68 cm (Figure 6).

Due to occupational constraints, the patient was unable to return to low altitude promptly. During this period, management consisted of daily low-flow oxygen therapy for over 16 h, strict limitation of physical activity, and primarily bed rest. After 24 days of this regimen, a follow-up echocardiogram was performed. The study revealed mild progression of right heart enlargement, characterized by increased dimensions of the right atrium and right ventricle (right ventricular transverse diameter 39 mm; right atrial diameter 46 mm). The estimated sPAP was approximately 47 mmHg, and the calculated mPAP was 31 mmHg.

#### Differential diagnosis considered the following etiologies

Chronic thromboembolic pulmonary hypertension (CTEPH): Patients had no risk factors for deep vein thrombosis, and D-dimer was normal. Pulmonary hypertension due to left heart disease: No history, symptoms, or echocardiographic evidence supported left heart disease, thus excluded. Pulmonary hypertension due to lung diseases and/or hypoxia: Chest CT showed no emphysema, interstitial lung disease, or masses, excluding these factors.

Based on the patients’ profile (young males), history of high-altitude residence, progressive hypoxic symptoms, objective findings of right heart enlargement and pulmonary hypertension, and exclusion of other common secondary causes, the final diagnosis was HAPH.

## Discussion

This report describes two cases of severe HAPH in previously healthy young males following long-term exposure to an environment above 4,300 m. Traditionally, HAPH has been viewed as a slowly progressive disease, more commonly observed in middle-aged and elderly long-term migrants to high altitudes (8, 9). The particular significance of our cases lies in the fact that these were young males who developed rapid progression to right heart dilation and decompensation within a relatively short exposure period. This suggests that high-altitude exposure can induce rapid and severe pathological changes even in young populations. The clinical features, including significant hypoxemia, secondary polycythemia, and echocardiographic findings of right atrial and ventricular enlargement with moderate tricuspid regurgitation, all indicate markedly increased right ventricular pressure overload, potentially advancing to a decompensated stage. It is noteworthy that the clinical courses of the two patients diverged significantly. Following transfer to a low-altitude environment without any targeted intervention, the sPAP in Case 1 gradually decreased and normalized within 1 month. In contrast, despite prolonged oxygen therapy and strict bed rest at high altitude, Case 2 showed no significant reduction in sPAP, and right heart enlargement persisted.

The core pathophysiological mechanisms of HAPH involve hypoxia-induced sustained pulmonary vasoconstriction and vascular remodeling (10). The low-pressure, hypoxic environment acts as the initiating factor, promoting the generation of oxygen free radicals, which triggers oxidative stress leading to vascular endothelial cell injury and dysfunction (11, 12). As a compensatory response, the body increases erythropoiesis to improve oxygen delivery. However, excessive polycythemia increases blood viscosity, further increase in pulmonary vascular resistance and right ventricular afterload (13). Second, hypoxia inhibits potassium channels in pulmonary vascular smooth muscle cells, inducing membrane depolarization and promoting calcium influx, ultimately leading to increased intracellular calcium levels and vasoconstriction (14). This precapillary constriction effectively shields the capillary bed from hypoxiainduced pulmonary hypertension by restricting the pressure upstream, thereby maintaining a relatively low hydrostatic pressure in the downstream pulmonary capillaries. It serves as the body’s most crucial immediate defense mechanism against excessive fluid filtration from pulmonary vessels and the prevention of pulmonary edema (15, 16). However, under the combined effects of sustained hypoxia and individual susceptibility, the pathological process of vascular remodeling is initiated. This process is characterized by aberrant proliferation of the extracellular matrix, pulmonary arterial endothelial cells, and smooth muscle cells, ultimately leading to thickening of the pulmonary arterial wall and luminal narrowing (14). When pathological vascular wall thickening and stiffening reach a certain severity, the precapillary vessels can no longer maintain effective contractile tension, and their “gating” function becomes compromised or dysfunctional. Consequently, the significantly elevated pulmonary arterial pressure is transmitted directly to the pulmonary capillary bed, causing a sharp rise in capillary hydrostatic pressure (5, 17). When this uncontrolled hydrostatic pressure exceeds the physiological tolerance of the pulmonary capillary wall and the alveolar-capillary barrier, it induces barotrauma (or mechanical injury), significantly increasing its permeability. This allows protein-rich fluid and red blood cells to leak into the alveolar space in the absence of prior inflammation, leading to the development of secondary high-pressure pulmonary edema and severely compromising gas exchange function (18–20). The consequent continuous rise in right ventricular afterload may ultimately progress to right heart failure and death (21, 22).

Pulmonary hypertension serves as the foundation for the development of HAPE, but alone it is insufficient to cause the disease. An imbalance in alveolar fluid clearance represents another critical pathogenic mechanism (23). The quantity of alveolar fluid is determined by both the leakage of fluid from pulmonary vasculature, primarily associated with hypoxic pulmonary hypertension, and the rate of fluid reabsorption by the alveolar epithelium, which is largely governed by sodium transport within these cells (24). Thus, hypoxia-induced dysregulation of pulmonary fluid balance is central to driving this pathological process. Disturbances in nitric oxide (NO) metabolism contribute significantly to impaired vasodilation (25). In the hypoxic high-altitude environment, elevated levels of asymmetric dimethylarginine (ADMA) competitively inhibit endothelial nitric oxide synthase (eNOS) activity, thereby directly reducing the synthesis of endogenous NO (26). Studies have shown that while pulmonary NO release increases in healthy individuals upon high-altitude exposure, it remains unchanged in individuals susceptible to HAPE, suggesting an inherent impairment (27). Furthermore, hypoxia-induced oxidative stress promotes the generation of free radicals, which in turn accelerate the breakdown of NO, further diminishing its bioavailablity (28). These mechanisms collectively lead to a marked reduction in NO, culminating in the onset and progression of pulmonary hypertension.

Individual susceptibility to high altitude varies significantly, underpinned by complex genetic adaptation mechanisms. Genome-wide association studies have identified significant genetic variations in genes related to oxygen sensing (e.g., EGLN1), energy metabolism, and mitochondrial homeostasis (e.g., EPAS1) associated with high-altitude adaptation (29). Animal studies suggest a significant correlation between the expression of the pulmonary arterial hypertension-related gene BMPR2 and the hypoxia-sensing gene HIF1A during short-term high-altitude acclimatization (30). Furthermore, epigenetic regulation is also recognized as playing an important role in the development and progression of pulmonary hypertension (31). In fact, the variation in the ability to maintain pulmonary fluid homeostasis and tolerate hypoxia among individuals ascending from sea level to high altitude may originate from a structural and functional divergence of the blood-air barrier (32).

A limitation of this study is that right heart catheterization (RHC), the current gold standard for diagnosing pulmonary hypertension (33). We adopted the current echocardiographic diagnostic criterion for high-altitude pulmonary hypertension, which is defined as mPAP ≥ 30 mmHg at rest in high-altitude areas, calculated by the formula: 0.61 × sPAP + 2 mmHg. This contrasts with the diagnostic threshold of mPAP ≥ 25 mmHg used in low-altitude regions (34, 35). The estimated sPAP in both patients exceeded 55 mmHg, meeting the criteria for PH. Although the 6-min walk distance is valuable for assessing prognosis in PH patients (36), it is important to note that young patients may still have severe PH even with a walk distance exceeding 500 m (37), highlighting that functional assessment must be interpreted alongside imaging and hemodynamic parameters. After excluding other common causes of PH, such as chronic thromboembolic PH, left heart disease-associated PH, and lung disease-related PH, the diagnosis of HAPH was supported.

These cases carry important clinical implications. Firstly, for young individuals presenting with exertional dyspnea, chest pain, or syncope after returning from high altitudes, clinicians should maintain a high index of suspicion for HAPH, even in those with no prior medical history. Of note, early disease may manifest solely as exercise-induced pulmonary hypertension, where pulmonary pressures rise abnormally during exertion but are normal at rest (38, 39). Therefore, early screening with pulse oximetry, arterial blood gas analysis, and echocardiography is crucial to avoid diagnostic delay. Secondly, beyond the cardiovascular system, chronic hypoxic exposure can adversely affect the nervous system, leading to cognitive decline and an increased risk of depression (40, 41), potentially linked to systemic oxidative stress and inflammatory responses. This underscores the need for a comprehensive, multi-system assessment in HAPH patients. Regarding treatment, relocation to lower altitude remains the most effective intervention (42). Currently approved therapies for pulmonary arterial hypertension (PAH) target three central pathogenic pathways: the endothelin, prostacyclin, and NO pathways. Corresponding treatment strategies can be categorized into two main approaches: the use of endothelin receptor antagonists (ERAs) to inhibit vasoconstriction, and the promotion of vasodilation via agents such as prostacyclin analogs, prostacyclin receptor agonists, phosphodiesterase type 5 inhibitors (PDE5i), or soluble guanylate cyclase (sGC) stimulators (43, 44). Acetazolamide, a carbonic anhydrase inhibitor, represents a potential strategy for drug repurposing. It acts by inducing mild metabolic acidosis, which stimulates central and peripheral chemoreceptors, significantly increasing minute ventilation. This leads to improved arterial oxygen saturation, subsequent pulmonary vasodilation, and ultimately a reduction in pulmonary vascular resistance and pulmonary arterial pressure (45). However, in patients who already present with dyspnea and are nearing respiratory muscle fatigue, acetazolamide may exacerbate breathing effort and potentially precipitate respiratory failure (46). Traditional Chinese medicines and their natural active components have also demonstrated potential in alleviating HAPH symptoms (44). Studies have shown that natural compounds such as resveratrol, dihydromyricetin, and salidroside can effectively inhibit the abnormal proliferation of pulmonary artery smooth muscle cells and mitigate oxidative stress and right ventricular remodeling, thereby offering new directions for future individualized therapy (47, 48). It is important to emphasize that hypoxic pulmonary vasoconstriction represents a physiological compensatory reflex that optimizes ventilation/perfusion matching during acute hypoxic exposure. Indiscriminate suppression of this reflex in the early stages of high-altitude exposure may therefore disrupt physiological adaptation and pose certain risks. However, when this response persists and progresses into pathological pulmonary vascular remodeling, leading to sustained pulmonary hypertension, the use of targeted pharmacological agents to induce pulmonary vasodilation becomes a rational treatment strategy (15, 49).

In summary, this report of two severe HAPH cases in young males highlights the significant threat that high-altitude environments can pose to the cardiovascular health of susceptible individuals. Enhancing clinical vigilance, improving early recognition, advancing comprehensive management, and deepening the exploration of genetic predispositions and pathogenesis are all critical for safeguarding the health of those living and working at high altitudes.

## Data Availability

The original contributions presented in this study are included in this article/supplementary material, further inquiries can be directed to the corresponding authors.
